# Supplementary material for: Aerosol Delivery of Palivizumab in a Neonatal Lamb Model of Respiratory Syncytial Virus Infection
Source: Viruses. 2023 Nov 19;15(11):2276. doi: 10.3390/v15112276 (PMC10675108; doi:10.3390/v15112276)
Supplement: Supplementary file 1 [file viruses-15-02276-s001.zip › viruses-2708087-supplementary.pdf]

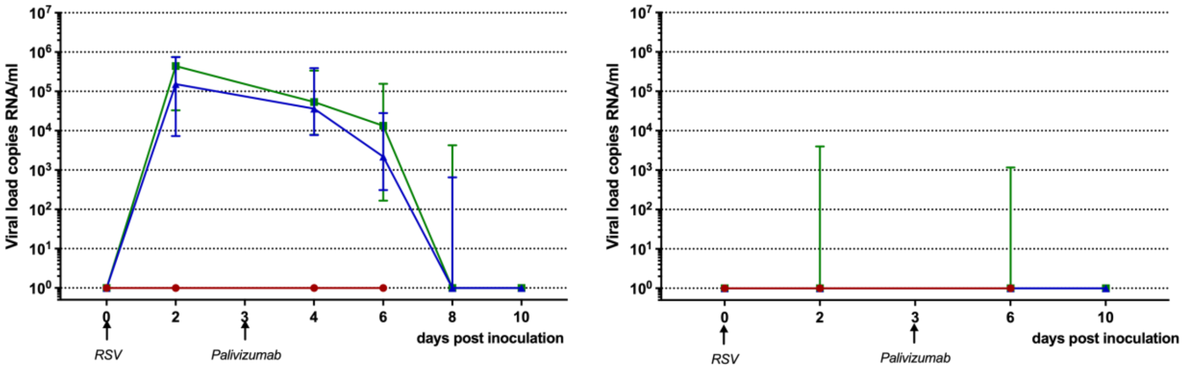


**Figure S1.** – Comparison of RSV load in nasal swabs and BALF samples. A] Nasopharyngeal (NP) swabs and B] bronchoalveolar lavage fluid (BALF) pellet samples viral load is displayed as a median of all lambs in each group. Red circles = Control Group 0, Green squares = RSV/untreated Group 1a,b, Blue Triangles = RSV/palivizumab Group 2a,b; data presented as median ± IQR values.
